# Shape-dependent ordering of gold nanocrystals into large-scale superlattices

**DOI:** 10.1038/ncomms14038

**Published:** 2017-01-19

**Authors:** Jianxiao Gong, Richmond S. Newman, Michael Engel, Man Zhao, Fenggang Bian, Sharon C. Glotzer, Zhiyong Tang

**Affiliations:** 1 Chinese Academy of Science (CAS) Key Laboratory of Nanosystem and Hierarchical Fabrication, CAS Center for Excellence in Nanoscience, National Center for Nanoscience and Technology, Beijing 100190, China; 2 Department of Chemical Engineering, University of Michigan, Ann Arbor, Michigan 48109, USA; 3 Biointerfaces Institute, University of Michigan, Ann Arbor, Michigan 48109, USA; 4 Shanghai Synchrotron Radiation Facility, Shanghai Institutes of Applied Physics, No. 239, Zhangheng Road, Shanghai, 201204, China; 5 Department of Materials Science & Engineering, University of Michigan, Ann Arbor, Michigan 48109, USA

## Abstract

Self-assembly of individual building blocks into highly ordered structures, analogous to spontaneous growth of crystals from atoms, is a promising approach to realize the collective properties of nanocrystals. Yet the ability to reliably produce macroscopic assemblies is unavailable and key factors determining assembly quality/yield are not understood. Here we report the formation of highly ordered superlattice films, with single crystalline domains of up to half a millimetre in two dimensions and thickness of up to several microns from nanocrystals with tens of nanometres in diameter. Combining experimental and computational results for gold nanocrystals in the shapes of spheres, cubes, octahedra and rhombic dodecahedra, we investigate the entire self-assembly process from disordered suspensions to large-scale ordered superlattices induced by nanocrystal sedimentation and eventual solvent evaporation. Our findings reveal that the ultimate coherence length of superlattices strongly depends on nanocrystal shape. Factors inhibiting the formation of high-quality large-scale superlattices are explored in detail.

## Abstract (web summary)

Ordered self-assembly is a promising way to realize collective properties in nanocrystals, but reliable routes to such macroscopic structures are missing. Here the authors make cm-scale ordered superlattice films from gold nanoclusters, correlating film properties with the shape of the building blocks

Nanocrystal self-assembly mimics the organization of atoms into a crystal and attracts intense research interest to create nanostructured materials^1^,^2^,^3^,^4^,^5^,^6^,^7^,^8^. A central aim is to combine the physical properties of individual nanocrystal building blocks with novel functionalities arising from the collective behaviour of nanocrystals into ordered superlattices, for example exploitation of the strong surface plasmon resonance^9^,^10^,^11^,^12^,^13^,^14^ and collective oscillations of free electrons in noble metal nanocrystal superlattices^10^,^15^. Evidently, the fabrication of superlattices with order over sufficiently long distances relative to individual nanocrystal size is crucial for device applications. State-of-the-art assembly techniques have produced superlattices of well-defined morphology (supercrystals) with several microns in diameter via evaporation from 30 to 70 nm gold nanocrystals^16^. Particularly large superlattices are achievable via sedimentation, as recently demonstrated for 100–300 nm silver nanocrystals^17^. Utilizing the lithographically obtained capillary channel on substrates, about 100 layer thick three-dimensional superlattices have been obtained from both 5.5 nm CdSe and 8.0 nm Fe_2_O_3_ nanocrystals^18^. Distinct from conventional crystallization of dimensionless atoms, nanocrystals generally have characteristics of size^3^, shape^19^ and interparticle interaction^20^. Understanding the role of these factors for superlattice formation is important to achieve control over the assembly process.

Because of the rapid development of shape-controlled synthesis of nanocrystals^21^,^22^,^23^, shape has emerged as a particularly important factor for self-assembly^16^,^24^,^25^,^26^,^27^,^28^. One broad category are close-packed superlattices, usually from large nanocrystals with comparably narrow ligand shells, including rods, triangular plates and polyhedra^14^,^15^,^16^,^17^,^29^. Theoretical predictions^19^,^28^,^30^,^31^ and experimental observations^17^,^25^,^27^,^32^,^33^ agree that the arrangement of nanocrystals in close-packed superlattice depends crucially on their shape. However, the role of shape on the assembly process (that is, how do nanocrystals transform from their disordered starting configuration to the ordered superlattice) and the quality of the assembly product (that is, how does shape affect the long-range order in superlattices) remains poorly understood. Here we target to address these two open questions. We perform growth experiments of various types of gold nanocrystals into large-scale superlattices and analyse the crystallographic quality and shape dependency of the resulting superlattices. We emphasize the importance of shape by avoiding using small nanocrystals with relatively long ligand chains as building blocks, which can stabilize the open lattices because of the combination of shape and ligand effects^34^,^35^,^36^,^37^. Details of the formation process of the superlattices are investigated through computer simulations.

## Results

### Formation of gold nanocrystals superlattices

We synthesized single crystalline gold nanocrystals with four of the most common shapes (Supplementary Fig. 1a–d) using the seed-mediated growth method^22^. Spherical nanocrystals (diameter 40 nm), obtained by etching nanorod precursors, are grown into polyhedra by varying the Wulff shape of nanocrystalline gold through controlling solvent chemistry. Polyhedra are characterized by their edge length: octahedra (74 nm), cubes (69 nm), three sizes of rhombic dodecahedra, small (sRD, 33 nm), medium (mRD, 45 nm) and large (lRD, 74 nm). Each set of nanocrystals is significantly monodisperse with size dispersity equal to or <5%. As-synthesized gold nanocrystals with concentration of 10^−9^ mol l^−1^ were dispersed in cetylpyridinium chloride (CPC) solution of controlled concentration. CPC molecules attach to the nanocrystal surface and offer short-range steric repulsion to counterbalance van der Waals attraction, preventing the nanocrystals from uncontrollable aggregation. We deliberately chose relatively large nanocrystals and comparably small spacer molecules to minimize the effect of ligands on nanocrystal interaction.

The gold nanocrystals were allowed to assemble in tilted glass cuvettes at room temperature in an undisturbed environment by densification via sedimentation. As the monodisperse gold nanocrystals gradually settled to the bottom of the cuvette over the course of one day for the largest nanocrystals and up to a few days for the smallest nanocrystals, local nanocrystal concentrations and collisions increased, and self-assembled superlattices emerged slowly. In parallel with sedimentation, but on the slower time scale of a week, evaporation of the water contributed to densification by reducing the volume available to the nanocrystals. The ordered superlattices dried during the final stage of the solvent evaporation and remained in the cuvette as a bulk film with thickness ranging from a few nanocrystal layers (∼100 nm) to hundreds of nanocrystal layers (∼10 μm). Films were peeled off from the cuvette wall using conductive carbon adhesive tape. The final assembly product is a free-standing film of macroscopic dimensions composed exclusively of highly ordered CPC-stabilized gold nanocrystals (Supplementary Fig. 1e,f).

**Figure 1:**
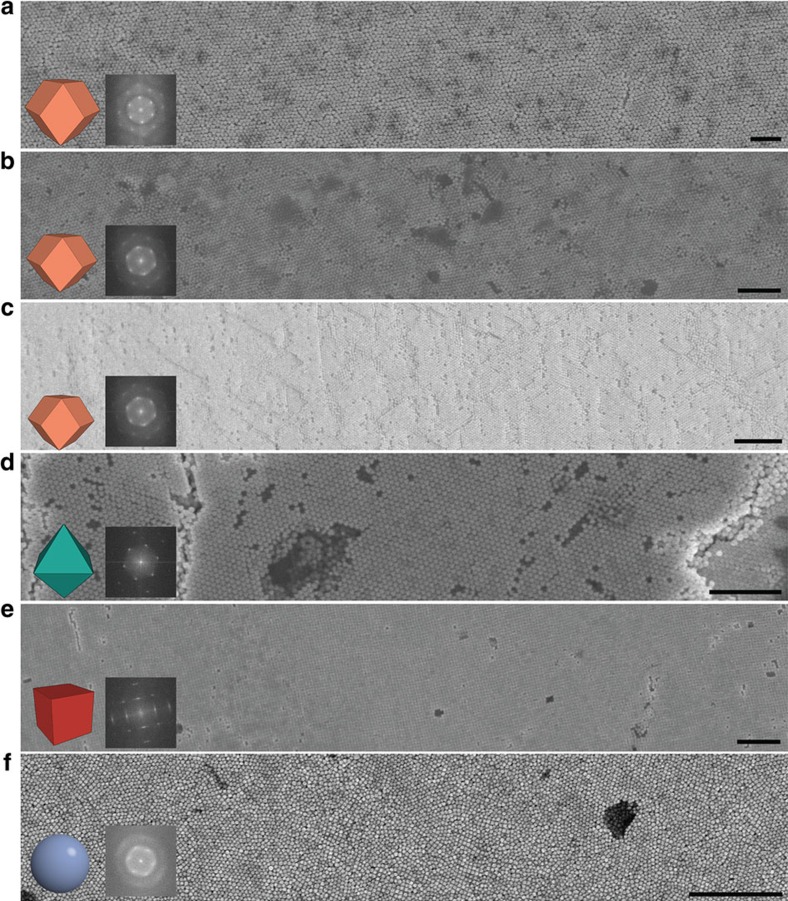
Large-scale SEM images of superlattices using gold nanocrystals. The nanocrystal shapes are (**a**) large (lRD), (**b**) medium (mRD), (**c**) small (sRD) rhombic dodecahedra, (**d**) octahedra, (**e**) cubes and (**f**) spheres. The view is chosen perpendicular to the bottom of the superlattice surface, which was in contact with the glass cuvette before being peeled off. The particle shape and an image calculated from a fast Fourier transform of the SEM image are shown as insets. As confirmed by sharp spots in the diffraction images, all superlattice films with exception of that formed from spheres are single domains across the SEM image. Scale bar, 1 μm.

Scanning electron microscopy (SEM) images of superlattices assembled with each of the six sets of nanocrystals are shown in Fig. 1. As expected, the geometric arrangement of the nanocrystals depends on building block shape^19^. Except for spheres, all sets of nanocrystals formed films visibly ordered over a distance of a minimum of several tens of microns. For spheres, diffuse rings rather than sharp spots are discernible in the fast Fourier transform (FFT) image (Fig. 1f, inset), which means that they exhibit no long-range ordering. The largest ordered areas are several microns in size (Supplementary Fig. 2), which is significantly smaller than the size of ordered domains in superlattices assembled from rhombic dodecahedra, octahedra and cubes. We also observe that superlattice orientation is influenced by the cuvette wall. Cubes and octahedra contact the wall with one of their planar facets to achieve layers of dense packing. Rhombic dodecahedra achieve a higher in-plane packing density by having a vertex in contact with the wall. Just like in crystallization of atoms and molecules at a boundary or interface, the cuvette wall introduces some point defects and line defects in bottom surface layers visible in Fig. 1. However, as demonstrated below, the surface defects do not extend into the inner layers of the superlattice and ordering is maintained over large areas.

### Superlattice quality and crystallographic order

**Figure 2:**
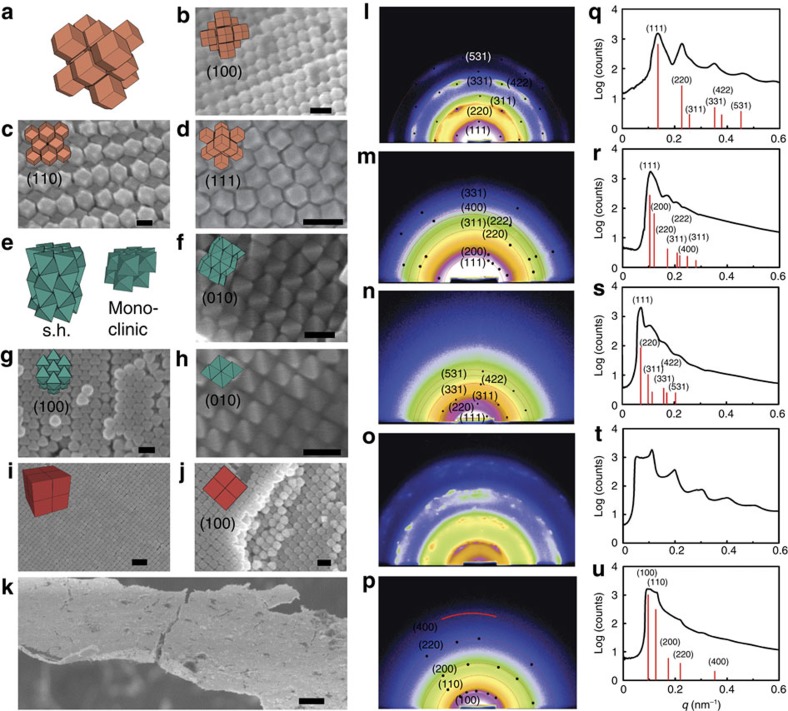
Analysis of nanocrystal superlattices in using SEM and using SAXS. (**a**–**d**) Rhombic dodecahedra form a face-centered cubic superlattice visible along different projection directions. (**e**–**h**) Octahedra are found predominantly in two crystallographically distinct superlattices: simple hexagonal, s.h., (**e**–**g**) and monoclinic (**e**,**h**). (**i**,**j**) Cubes assemble into a simple cubic lattice. (**k**) A macroscopic view shows a complete superlattice film. (**l**–**p**) Two-dimensional and (**q**–**u**) radially averaged SAXS images of assembly products exhibit clear diffraction spots and peaks, respectively, highlighting long-range order in superlattices of sRD (**l**,**q**), mRD (**m**,**r**), lRD (**n**,**s**), octahedra (**o**,**t**) and cubes (**p**,**u**). For (**l**–**p**) the data are shown in data on a logarithmic intensity scale and for (**q**–**u**), the vertical axes represent the logarithmic intensity. Peaks are not indexed for the octahedron superlattice (**o**,**t**), because the crystal structure is not unique. See Supplementary Movie 1 for more information. Scale bars, (**i**) 200 nm; (**k**) 10 μm; all others are 100 nm.

To analyse the coherency of superlattices over large scales, small-angle x-ray scattering (SAXS) measurements (Supplementary Fig. 3) were carried out at the Beijing Synchrotron Radiation Facility and Shanghai Synchrotron Radiation Facility, of which the diffraction patterns could demonstrate the crystalline quality of superlattices (see Methods for more detail for SAXS). All assemblies from polyhedra exhibit clear diffraction spots (Fig. 2l–p; Supplementary Figs 4 and 5) indicating three-dimensional long-range order throughout the samples. In agreement with the visual inspection in Fig. 1, there are no diffraction spots discernible for gold sphere assemblies (Supplementary Fig. 6). This confirms that the typical domain size for our sphere superlattices is significantly smaller than the sample dimensions.

The crystallographic structure of the superlattices can be determined if long-range order is present. SEM images confirm the expected close-packing of rhombic dodecahedra into the face-centered cubic (f.c.c.) lattice^38^. Each rhombic dodecahedron contacts with twelve neighbours facet-to-facet (Fig. 2a–d) and diffraction peaks are indexed to the f.c.c. lattice (Fig. 2q–s). Although all sets of rhombic dodecahedra with varying size (sRD, mRD and lRD) show clear diffraction peaks, superlattices of the smallest particle (sRD) exhibit the sharpest and most intense diffraction spots of a single crystalline character. This suggests that despite the relatively smallest size of sRDs, their ordered domains are the largest. To further confirm the single crystallinity and nanocrystal orientation, we performed rotating SAXS and single crystal diffraction for sRD superlattices (Supplementary Fig. 5)^34^,^35^. The <100>, <110> and <111> orientation of superlattice and the orientation of the gold nanocrystal are identified respectively, which demonstrated the single crystallinity of superlattice and consistent orientation of nanocrystals in side. It should be noted that the smallest beam size used here is as large as 0.5 × 0.5 mm^2^, which may cover several domains and disordered parts, leading to polycrystalline diffraction spots or powder-like diffraction features in those samples with single crystal domain smaller than the beamline area. Under normal circumstances the superlattice domain size can be obtained using the Scherrer formula from measurements of the full width at half-maximum intensity of a diffraction peak^25^,^39^,^40^. Here, however, peak widths are comparable to the resolution limit of the beamline, 1.55 μm. As this is much smaller than the domain sizes directly observable by SEM (Fig. 1), the SAXS peak width analysis can only provide a lower bound. Instead, we rely on visual inspection over macroscopic distances. For sRD, we find single crystalline superlattice domains larger than 0.5 mm in extent (Supplementary Fig. 7) by comparing the crystal orientation along the sample. Order persists over many thousands of layers (Supplementary Figs 8–10).

SAXS patterns of octahedron superlattices have well-defined peaks that cannot be assigned to a single lattice type (Fig. 2o,t; Supplementary Fig. 11). Indeed, as the SEM analysis reveals, octahedron nanocrystals frequently assemble in two crystallographically distinct stacking modes of close-packed hexagonal layers, a base-centered monoclinic superlattice with a two-particle unit cell (Fig. 2e,h) and a simple hexagonal superlattice (Fig. 2e–g). A third structure, the densest packing of octahedra with density 18/19, appears occasionally as well. This lattice, known as the Minkowski lattice [Fig f3](Fig. 4g; Supplementary Fig. 12), was previously reported with ideal hard octahedra in simulation^30^,^41^. The simple hexagonal superlattice and the Minkowski superlattice have previously been assembled from octahedral silver^17^. Inside monoclinic and simple hexagonal assemblies six nanocrystals surround each octahedron in partial facet-to-facet contact to form dense packing layers (Fig. 2e). The difference between the two lies in the orientation and the relative translational offset of the octahedra in adjacent layers. In the monoclinic packing all octahedra have identical orientation and adjacent layers are offset such that a vertex touches the center of an edge (Fig. 2h). In the simple hexagonal arrangement octahedra in adjacent layers have opposite orientation and are in perfect facet-to-facet contact (Fig. 2f,g). Among the possible polymorphs for the octahedron superlattices we observe the simple hexagonal arrangement most frequently. Visual inspection shows that the size of ordered domain of octahedron superlattice typically reaches only 100 microns (Supplementary Fig. 13), much smaller than sRD, mRD and lRD domains. This indicates that shape rather that size determines the quality of the assembly product.

It is evident from SEM images that cubes arrange into a simple cubic lattice (Fig. 2i,j). SAXS data demonstrates that it is more challenging to achieve order with cubes over large areas as compared with rhombic dodecahedra and octahedra (Fig. 2p,u). The largest domain size visible in large-area SEM images (Supplementary Fig. 14) is about 30 microns in diameter. Delocalized vacancies, a characteristic type of disorder predicted with simulation^42^, are frequently found in the cube superlattices.

### Nucleation and growth of superlattices in simulation

Our experimental results are evidence that nanocrystal shape has a strong effect on superlattice quality. To understand the origin of this effect, we performed Monte Carlo computer simulations of the self-assembly process, which can help to predict/study the assembly process and figure out the important factors affecting the results through parameters' control. At the ordering transition, the nanocrystals interact with one another via van der Waals attraction of the gold cores and CPC ligands, depletion attraction caused by unabsorbed CPC molecules, and electrostatic repulsion of CPC ligands^20^. In our experiments these interactions are deliberately tuned to be weak as to prevent aggregation in solution and produce contact forces that act over short distances of only a few nanometres. Our nanocrystal systems can therefore be described well by the hard particle model during sedimentation^19^,^43^,^44^. This model maintains the anisotropy and orientational dependence of the excluded volume but ignores other effects. Hard polyhedra strive to align along their facets to maximize entropy and minimize free energy, a phenomenon known as directional entropic forces^25^,^45^. Because entropic forces increase in strength and range with facet dominance^38^, polyhedral nanocrystals should be superior candidate building blocks for forming high-quality superlattices compared with spherical nanocrystals, as borne out by our experiments. Since it is not possible to reach experimental system sizes (>10^7–12^ nanocrystals) or the time span of an assembly experiment (>10^5^ s) with present or near-future computer resources, we extrapolate from small systems and short simulation times compared with experimental conditions.

**Figure 3 f3:**
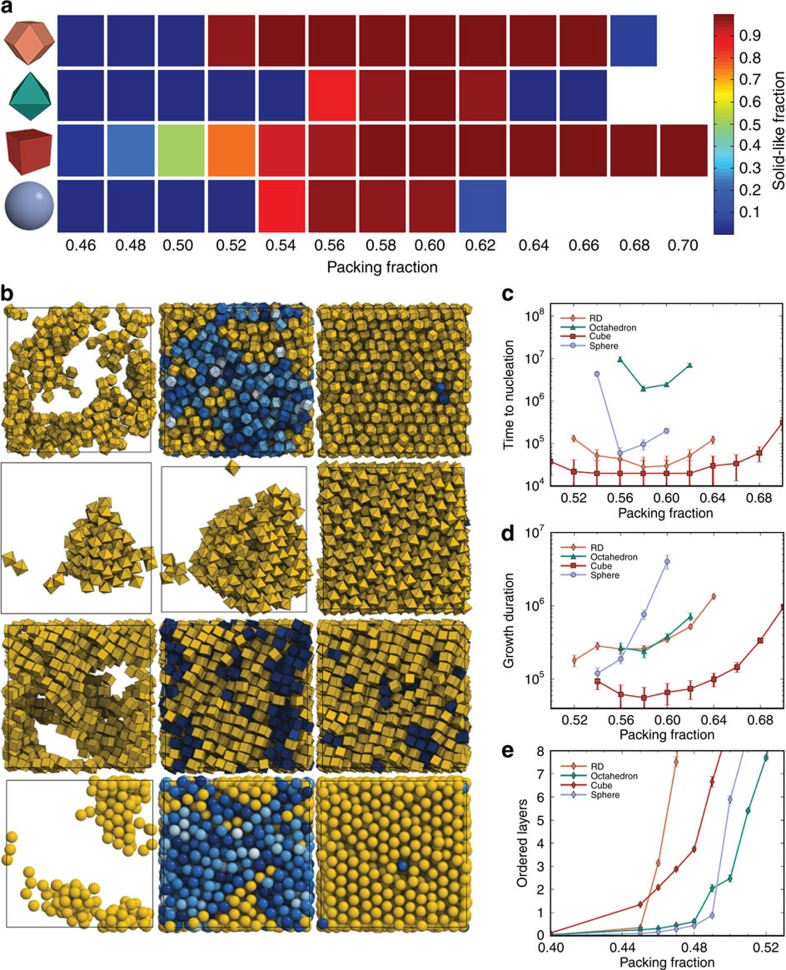
Crystallization of nanocrystal superlattices in Monte Carlo simulations of hard particles. (**a**) Density range where homogenous nucleation and growth occurs for each particle shape using isochoric simulations starting from configurations rapidly compressed to a selected packing density. Rectangles in a density versus shape grid are coloured by the calculated system-average local order for rhombic dodecahedra, octahedra, cubes and spheres. Low values (blue) indicate disordered local structure (fluid), while high values (red) indicate crystalline order. Empty cells demarcate the regions beyond random close packing. Each data point represents the average of ten runs over 50 million Monte Carlo cycles. (**b**) Early, middle and late stage growth of homogeneously nucleating superlattices for rhombic dodecahedra, octahedra, cubes, and spheres. Densities are selected to represent a typical Monte Carlo trajectory for the particular shape. Crystallization may result from several nuclei. (**c**) Time to nucleate, as measured by the number of Monte Carlo cycles for the system to reach 20% crystallinity. (**d**) Time to grow, as measured by the time required to advance from 20 to 80% crystallinity. (**e**) Number of ordered layers equilibrated near hard boundaries at densities lower than where homogeneous nucleation occurs. The error bars in (**c**–**e**) correspond to the standard error of the mean.

We first study the effect of nanocrystal shape on homogenous nucleation. Ordering requires a critical density to be thermodynamically favourable but becomes kinetically inaccessible if density is too high. In experiment this means there is a time window during sedimentation in which density is sufficiently high to form superlattices, but low enough to still anneal defects. We estimate this time window by the density range over which a given system of nanocrystals exhibits dynamics. Figure 3a summarizes, for each shape, the density window for superlattice formation in simulation. Notably, each shape orders robustly and rapidly at packing densities near 56%. However, the density ranges over which assembly and annealing of defects are observed vary significantly. Cubes and rhombic dodecahedra order reliably over a wide range of densities, while octahedra and spheres require fine-tuning for assembly to occur.

Difference in the assembly behaviour of shaped nanocrystals affects the quality of as-formed superlattices. Representative early, middle and late time formation of superlattices for each shape is illustrated in Fig. 3b and time series of growth are shown in Supplementary Movie 2. We compare the time to nucleate (Fig. 3c, defined as the time for a system to reach 20% crystalline) and the growth duration (Fig. 3d, defined as the time to reach 80% crystalline from the nucleation time) measured in units of Monte Carlo cycles. As discussed in the Methods section, we estimate that one second of the assembly experiment corresponds to on the order of 10^4^ Monte Carlo cycles. We learn from Fig. 3b–d that rhombic dodecahedra rapidly form multiple nuclei from the metastable equilibrium fluid state at high enough density. Nuclei readily rearrange and anneal out defects, resulting in high-quality superlattices. In contrast, octahedra only show isolated (rare) nucleation events up to high densities. Nuclei grow quickly until crystallization is complete, but arrest sets in early. Cubes adopt local positional and orientational order almost immediately after reaching high enough density. There is neither an observable metastable fluid state nor a well-defined nucleation event for cubes, suggesting the nucleation barrier is negligible at almost all densities, where the cubic superlattice is stable. Spheres have a comparably narrow density window where nucleation occurs^46^. Multiple nuclei form rapidly above the critical density. Growth is also initially rapid, but defects anneal out significantly more slowly than for rhombic dodecahedra. Kinetic arrest sets in at lower density for spheres and octahedra than for rhombic dodecahedra and cubes.

In experiment, large-scale superlattice assembly proceeds via a sedimentation process wherein the density rises slowly near the cuvette wall. To investigate the entropic influence of the cuvette wall, we employ Monte Carlo simulations with a hard wall along one coordinate axis. Multiple crystalline layers form heterogeneously adjacent to the wall at significantly lower densities than those required for homogenous nucleation (Fig. 3e; Supplementary Fig. 15). For example, in the case of spheres, multiple ordered layers are already present at density 50%, while homogeneous nucleation starts around density 52% (ref. 46). Although diluted nanocrystal solutions magnify the effect of the wall, the wall affects the dominant superlattice structure only in the case of octahedra (Supplementary Fig. 16) but not for other shapes (Supplementary Figs 17–19), in agreement with prior simulations^47^.

### Factors affecting superlattice quality

Our results agree with earlier observations that sedimentation-driven assembly is more robust in yielding high-quality nanocrystal superlattices than experiments where density is under evaporative control^17^. Equilibrium order close to the wall could be reached within a few million Monte Carlo cycles, or on the order of seconds to minutes in experiment once density is high enough. So, in all cases, nucleation and growth is several orders of magnitude faster than the typical sedimentation time, which suggests that the assembly process is quasistatic with respect to changes in density. Neither homogenous nucleation nor heterogeneous nucleation is the rate-limiting factor. The quality of the superlattices for each polyhedron shape should be dominated by other yet-unexplored factors, which are elucidated below.

**Figure 4:**
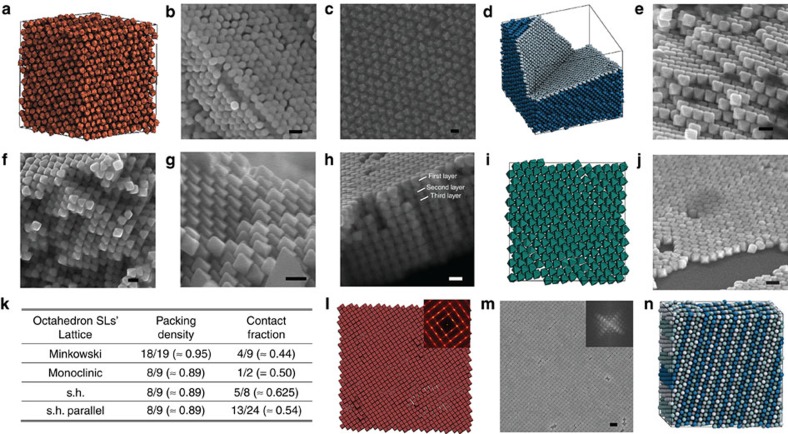
Structural details and factors affecting quality of nanocrystal superlattices. (**a**–**c**) Analysis of rhombic dodecahedra superlattices. In simulation we find a rotator (plastic) face-centered cubic phase at low density (**a**), and quenched in rotational disorder is also observed in SEM images (**b**,**c**). (**d**–**j**) Analysis of octahedron superlattices. In simulation Minkowski superlattice is found exclusively (**d**). In experiment we observe monoclinic superlattice (**e**,**f**), Minkowski superlattice (**g**), and simple hexagonal superlattice (s.h. parallel), in rare occurrences (**h**), alternation of octahedron orientations in layers of simple hexagonal superlattice is violated, as visible in the first and second layer of (**h**). Single layer of octahedron superlattice near walls in hexagonal packing in simulation (**i**) and experiment (**j**). Packing density and contact fraction of different lattices are compared in (**k**). (**l**,**m**) Analysis of cube superlattices. Simulation (**l**) and experiment (**m**) both show streaks in the fast Fourier transform images. (**n**) Monte Carlo simulations of hard spheres, typically resulting in stacking faults. Here is a typical final snapshot. Layers are coloured by ABC stacking sequence (A=white; B=light blue; C=blue). Scale bar, (**m**) 200 nm; all others are 100 nm.

The incomplete parallel orientation of rhombic dodecahedra found in some of the SEM images (Fig. 4b,c; Supplementary Fig. 20) likely originates from either a quenched-in rotator phase, which is stable at intermediate density (Fig. 4a), or a surface effect. At higher density, nanocrystal facets align because of directional entropic forces and ligand-induced attractive interactions, neither of which is available in spheres^25^,^38^,^48^. As a result, stacking defects, which frequently occur with spheres (Fig. 4n), are suppressed in rhombic dodecahedra. Furthermore, a second factor affects the quality of superlattices from spheres in experiment. In contrast to micron-sized spherical colloids, the sphericity of nanocrystals is limited by their natural tendency to develop crystalline facets. Altogether, practical limitations of sphere nanocrystal synthesis and the more efficient packing of rhombic dodecahedra in combination with localized orientational ordering contribute to the higher quality of superlattices assembled from rhombic dodecahedra than from spheres^49^.

For octahedra, the presence of competing phases poses further complexity not present in the other shapes. Homogenous nucleation simulations result in the Minkowski superlattice (Fig. 4d) or, with similar probability, the monoclinic superlattice. Whichever superlattice nucleates first grows and determines the final structure. The situation might be even more complicated in experiment and depends subtly on the proximity to the cuvette walls. We compare the packing density and contact fraction of four octahedron superlattice candidates in Fig. 4k. The contact fraction is defined as the area of the octahedron surface in contact with the surface of a neighbour. Furthermore, octahedron superlattices can have easy sliding modes, where ‘easy sliding' means that a column or plane can shift freely without encountering obstruction. While the Minkowski superlattice is fully rigid (Fig. 4g), the three other superlattices are stackings of dense hexagonal layers (Fig. 4i,j; Supplementary Fig. 16). These layers constitute easy sliding planes. In addition, the monoclinic superlattice has easy sliding columns, visible as grooves in the SEM images (Fig. 4e,f). Fracturing often occurs along easy sliding planes and columns (Supplementary Fig. 21). The dilemma of octahedra is apparent in the fact that three of the candidate structures each possess a different extremal property: (i) The Minkowski superlattice is the densest packing. It is the preferred phase under high pressure. (ii) The simple hexagonal superlattice has the highest contact fraction. It is the preferred phase if attraction dominates. (iii) The monoclinic superlattice has the highest number of easy sliding modes. It is an entropically preferred phase. Only the parallel variant of the simple hexagonal superlattice does not have an extremal property. This explains why it is never observed outside of isolated occurrences (Fig. 4h).

Cubes readily order over a wide packing density range, but tend to exhibit more positional disorder than other shapes because of delocalized vacancies^42^. A high amount of local shearing and distortion of the cubic lattice is apparent in both simulation (Fig. 4l) and SEM image (Fig. 4m), and is also easily visible in the FFT (insets) as linear streaks. This disorder is caused by the inability of cubes to lock together in the simple cubic lattice, allowing for columns and planes of cubes to collectively fluctuate and shear. The presence of localized disorder explains why the correlation length in the cube superlattice measured by the peak width in the SAXS data (Fig. 2p,u) is shorter than that of other polyhedral nanocrystals.

## Discussion

Self-assembly of nanoscale functional building blocks into high-quality macroscale superlattices is a crucial challenge to realize device scale applications. Using gold nanocrystals with four different shapes (cube, octahedron, rhombic dodecahedron and sphere) as building blocks, we have successfully constructed superlattices with coherent order over macroscopic scales. Through detailed analysis with SEM and SAXS, we reveal that crystalline quality of superlattices is strongly dependent on building block shape. Among the four shapes used, the rhombic dodecahedron is found to be optimal for the formation of high-quality superlattices, with the largest single crystalline domain exceeding half a millimetre in our experimental scale. This is the largest single crystal domain reported for nanoscale superlattices to date. Simulations of the formation process demonstrate that both nucleation and growth depend strongly on nanocrystal shape. Spheres, the most widely studied nanocrystal shape, assemble into the lowest quality superlattices. Octahedra superlattices order robustly but are affected by competing polymorphic equilibrium structures. The quality of cube superlattices is diminished by a high vacancy content, which cannot be overcome even by the extremely rapid assembly of cubes. Finally, both experiment and simulation points to rhombic dodecahedra to be overall the best assemblers among all four shapes and thus excellent candidates for superlattices with the highest quality and largest single crystalline domain size. Our study reveals that nanocrystals with different shapes undergo various ordering pathways, and multiple shape-related factors affect the final domain size. This work not only gives new insight into the conventional superlattice formation theory, but also offers unprecedented opportunity to construct high-quality nanocrystal superlattices through careful design and selection of nanocrystal shape.

The self-assembly process can be fully reproduced and understood in the scope of the hard particle model, which suggests that our study of shape factors is not merely limited to gold nanocrystals, but is inherent characteristic of the relationship between building block shape and self-assembly behaviour. Considering the abundant types of nanocrystals available, including semiconductors, oxides, metals and even hybrid nanostructures, our findings offer a basic guideline for building block selection. Beyond the four model shapes in this study, we expect particle shape to be used either solely or in combination with other factors in designing nanocrystals for self-assembly. Although here we focus the investigation on aspects of fabrication and the fundamental mechanism, we note that gold nanocrystals are a major class of plasmonic materials for device construction. Such large size and high-quality superlattices as reported in this work would be excellent systems for plasmonic crystals and metamaterials.

## Methods

### Nanocrystal synthesis

The synthesis of gold nanocrystals followed the modified seed-mediated method developed by us previously^22^. **Step 1:** gold nanorods were synthesized first. Approximately 0.25 ml of 10 mM HAuCl_4_ solution was added into a glass cuvette containing 9.4 ml of 100 M CTAB solution. Subsequently, 0.6 ml of freshly prepared ice-cold 10 mM NaBH_4_ solution was added immediately under stirring. After stirred for 3–5 min, the solution was kept under 30 °C for 2 h and used as seed solution for gold nanorod growth. To 10 ml of 100 mM CTAB solution, 0.5 ml of 10 mM HAuCl_4_ solution, 60 μl of 10 mM AgNO3 solution, 80 μl of 100 mM L-ascorbic acid solution and 24 μl seed solution were added subsequently and kept under 30 °C for 2 h to allow for the growth of gold nanorods. **Step 2:** gold nanocrystal spheres with average ovality below 4% (Supplementary Fig. 22) were obtained from chemical etching of the prepared gold nanorods. As-synthesized gold nanorod solution was centrifuged (12,000 r.p.m., 10 min) twice, first redispersed in water and then in 10 ml of 10 mM CTAB solution. Subsequently, 0.5 ml of 10 mM HAuCl_4_ solution and 0.1 ml of 100 mM ascorbic acid solution were added in sequence at 40 °C. Each addition was followed up by thorough mixing. The solution was kept at 40 °C for 1 h, followed by centrifugation at 12,000 r.p.m. for 10 min and redispersion in 10 ml of 10 mM CTAB. Afterwards, 0.2 ml of 10 mM HAuCl_4_ solution was added at 40 °C to further etch the obtained gold nanocrystals. After gentle mixing, the solution was left undisturbed to age at 40 °C for 12 h. Finally, the solution was centrifuged thrice (12,000 r.p.m., 10 min) and dispersed in 10 ml of 100 mM CPC solution. The resulting solution contained spherical gold nanocrystals for further synthesis of gold polyhedra and assembly. **Step 3:** polyhedral gold nanocrystals with varying shape were synthesized by using spherical gold nanocrystals as seeds. In each synthesis a certain amount of KBr solution, HAuCl_4_ solution, L-ascorbic acid solution and spherical gold nanocrystal solution were subsequently added into 5 ml of CPC solution at 30 °C in a water bath (Supplementary Table 1). The reacting solution was then left undisturbed at 30 °C for 2 h and terminated by centrifugation (10,000 r.p.m., 10 min). The final product was washed twice with water before observation under SEM.

### Assembly procedure

As-synthesized gold nanocrystals were used as building blocks without further purification. The nanocrystal concentration was adjusted to 10^−9^ mol l^−1^, while the surfactant concentration was set to (CPC)=1 mM for cubes and octahedra and (CPC)=0.1 mM for rhombic dodecahedra and spheres. Gold nanocrystal solution of 1 ml was then placed into a glass cuvette with square cross section. For control experiments testing boundary effects, the nanocrystal concentration was decreased by a factor of 100, while keeping other conditions the same. The glass cuvette was placed in a position at which its wall formed a 30° angle with the horizontal line to improve assembly. Subsequently, the solution was left in an undisturbed open-air environment for evaporation.

### Scanning electron microscopy characterization

For observation of individual nanocrystals (Supplementary Fig. 1), 3 μl of the washed nanocrystal solution was dropped onto a silicon substrate and allowed to dry at room temperature. For the observation of assembled films (all other SEM images), the samples were carefully pulled off the glass cuvette wall with conductive carbon adhesive tape. All images were obtained using a Hitachi S4800 Scanning Electron Microscopy operating at 10 kV. For the FFT of SEM images, the sharp spot arrays represent the periodicity of the area shown in corresponding SEM images, while each spot results from the Fourier transform of the certain parallel arrays of atoms aligned in the direction perpendicular to the vector from original points to the spot in the FFT image.

### Small-angle x-ray scattering characterization

SAXS measurements at wavelength λ=1.55 Å and bandwidth Δλ/λ=10^−3^ were carried out on the beamline 1W2A of the Beijing Synchrotron Radiation Facility. The beam area on the sample was 0.5 mm by 1 mm. The 1W2A area detector was a Mar 165 CCD camera with a pixel size of 80 μm by 80 μm and a total of 2,048 × 2,048 pixels. The sample to detector distance was 5,180 mm. A thin Pb strip was used as a beam-stop. The images were dark current corrected, distortion corrected, and flatfield corrected. Typical exposure time was 30 s. The rotating SAXS at wavelength λ=1.24 Å were carried out on the beamline BL16B1 of Shanghai Synchrotron Radiation Facility. The beam area on the sample was 0.5 mm by 0.5 mm. The sample to detector distance was 1,950 mm. Typical exposure time was 2 s. Other conditions were the same with SAXS above. Scattering images were calibrated and integrated using the Fit2D software. The diffraction spots were indexed according to the corresponding q-d relationship of different lattice types of superlattices.

Single crystal X-ray diffraction was performed at room temperature for sRD superlattice on a SuperNova diffractometer, using graphite-monochromated Mo Kα radiation (λ=0.71073 Å).

The incident X-ray can interact with the periodic self-assembly structures, leading to formation of diffraction spots on the two-dimensional detector, which is similar with single crystal X-ray diffraction in wide angle range. If the structures are polycrystalline or powder, the scattering pattern will turn out to be rings. Rotating the sample with respect to the incident X-ray can offer more complete structure information. Single crystal X-ray diffraction can help to figure out the orientation of nanocrystals inside the superlattices.

### Monte Carlo simulation

Nanocrystals were represented as hard polyhedra in an isochoric ensemble with periodic boundary conditions. Overlaps were detected via the Gilbert-Johnson-Keerthi distance algorithm in code used and tested in prior work^19^. Hard boundaries were implemented by preventing any vertex of the polyhedron from exiting the *z*-facing sides of the bounding box. Homogeneous nucleation simulations of *N*=2,048 polyhedra for Fig. 3a were equilibrate for 50 million Monte Carlo cycles. Heterogeneous simulations of *N*=4,096 polyhedra in Fig. 3e were equilibrated for 3 million Monte Carlo cycles. Each Monte Carlo cycle consisted of *N* randomly selected translation or rotation moves.

### Solid-liquid order parameter

Standard solid–liquid order parameters analysed the crystallized fraction of the system during each run. We first defined the bond orientational order parameter 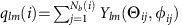 as a measurement of the local particle environment, where *N*_*b*_(*i*) is the number of neighbouring particles within a cutoff distance *r*_cut_ from particle *i*. For rhombic dodecahedra, octahedra, and spheres *r*_cut_ was chosen near the first minimum of the radial distribution function, for cubes analysis required using the first two coordination shells. The correlation between local environments of particles pairs is the scalar product 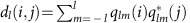. The number of sufficiently correlated local environments *ξ*(*i*) is the number of neighbours whose scalar product exceeds a threshold value *d*_*c*_. We defined solid-like particles as those with *ξ* exceeding a cutoff *ξ*_*c*_. Neighbouring solid-like particles were then clustered to identify nuclei. To calculate the number of ordered layers in wall simulations, the number of solid particles was normalized by the approximate number of particles in each layer, or *N*^2/3^. This estimate is exact for the simple cubic crystal, and within 10% for the other shapes. As for the order-parameter coloured figures (Fig. 3a), the solid-like particles were coloured yellow, and the liquid particles were coloured from a blue colour map ranging from darkest to lightest using the locally invariant spherical harmonic function 
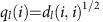
 (ref. 50). All parameters are given in Supplementary Table 2.

### Mapping Monte Carlo cycles onto experimental time

The relationship between simulation time and experimental time was estimated by comparing the mean square displacement measured from simulations in the fluid phase to the Brownian diffusion constant of the nanocrystals in water estimated using Stokes' law. This comparison is not exact, but sufficient to provide an order-of-magnitude estimation. A spherical gold nanocrystal with diameter *σ*=40 nm has Brownian diffusion constant 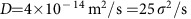 in water. We assumed that caging effects reduce nanocrystal diffusion by a factor of 100 at densities near solidification^51^. The comparison to mean square displacement from simulation then suggested there were ∼10^4^ Monte Carlo cycles per second of the experiment. The longest simulations over 50 million Monte Carlo cycles correspond to 1.5 h in experimental time. Simulations near hard walls over 3 million Monte Carlo cycles correspond to 5 min with the first crystalline layers appearing well within the first minute.

### Data availability

All relevant data are available from the authors on request.

## Additional information

**How to cite this article:** Gong, J. *et al*. Shape-dependent ordering of gold nanocrystals into large-scale superlattices. *Nat. Commun*. **8**, 14038 doi: 10.1038/ncomms14038 (2017).

**Publisher's note**: Springer Nature remains neutral with regard to jurisdictional claims in published maps and institutional affiliations.

## Supplementary Material

Supplementary Information Supplementary Figures and Supplementary Tables

Supplementary Movie 1 Small Angel X-Ray Scattering Data at Varying Intensity Ranges.

Supplementary Movie 2 Simulation Data of Time Series Growth of Superlattices.
