# Supplementary material for: Shape-dependent ordering of gold nanocrystals into large-scale superlattices
Source: Nat Commun. 2017 Jan 19;8:14038. doi: 10.1038/ncomms14038 (PMC5253678; doi:10.1038/ncomms14038)
Supplement: Supplementary Information — Supplementary Figures and Supplementary Tables [file ncomms14038-s1.pdf]

Supplementary Table 1. Synthesis conditions for the growth of polyhedral nanocrystals from spherical nanocrystals. Abbreviations are V: volume; C: concentration; oct: octahedron; sRD: small (33 nm edge length) rhombic dodecahedron; mRD: medium (45 nm) rhombic dodecahedron; lRD: large (74 nm) rhombic dodecahedron.

	**CPC**		**KBr**		**$HAuCl_4$**		**Ascorbic Acid**		**Seed**
	**V (mL)**	**C (mM)**	**V (μL)**	**C (mM)**	**V (μL)**	**C (mM)**	**V (μL)**	**C (mM)**	**V (μL)**
**cube**	5	100	50	100	100	10	15	100	200
**oct**	5	100	0	--	100	10	15	100	200
**sRD**	5	10	0	--	100	10	200	100	800
**mRD**	5	10	0	--	100	10	100	100	200
**lRD**	5	10	0	--	100	10	50	100	50

Supplementary Table 2. Parameter values for the order parameter analysis. Abbreviations are fs: frame spacing of Supplementary Video 2 in Monte Carlo cycles; ϕ: packing fraction; N: number of particles. The remaining symbols are defined in the method section.

Shape	fs	ϕ	N	l	d_c	r_{cut}	ξ_c	q_l
**rhombic dodecahedron**	50000	0.56	4096	6	16	2.2	6	[0.2, 0.7]
**octahedron**	50000	0.56	4096	6	16	1.8	6	[0.2, 0.7]
**cube**	10000	0.55	4096	8	18	2.2	12	[0.15, 0.35]
**sphere**	100000	0.54	4096	6	16	1.4	6	[0.2, 0.7]

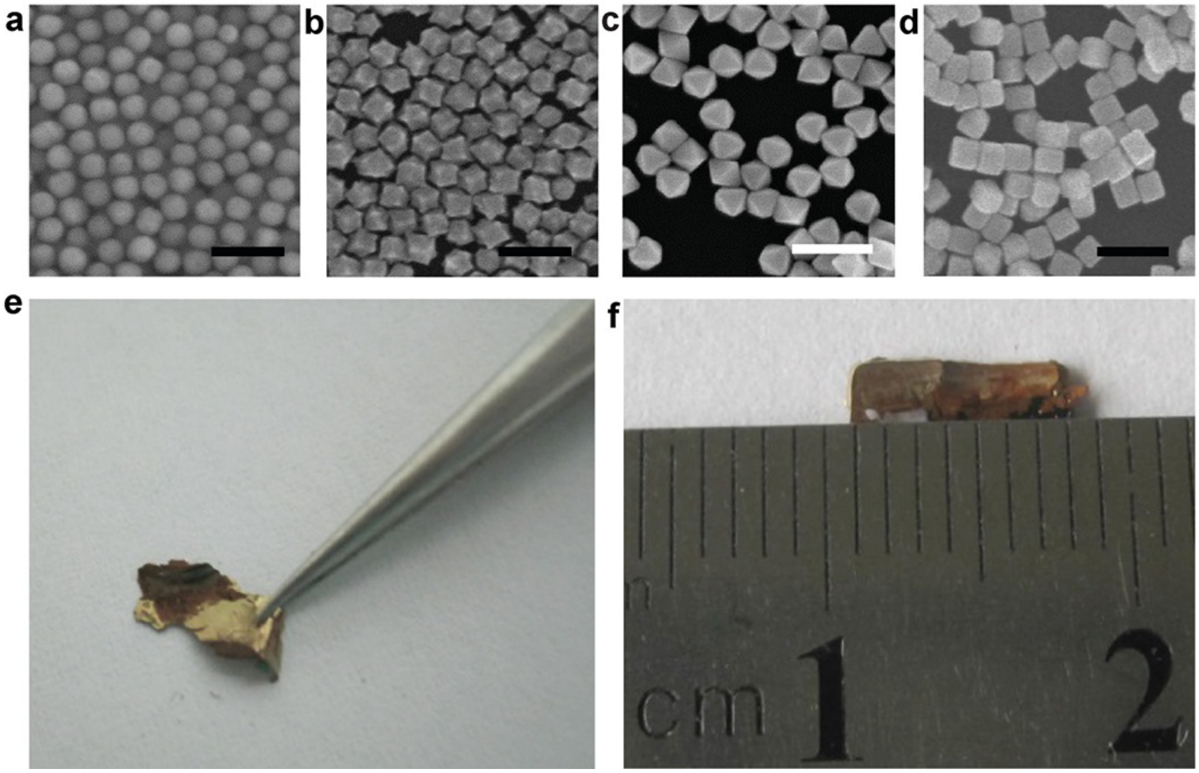

Supplementary Figure 1. SEM images of unassembled nanocrystals and the final assembly products. The gold nanocrystal shapes used in this study are (a) spheres, (b) rhombic dodecahedra, (c) octahedra, and (d) cubes. The edge length of the particles is a few tens of nm. (e,f) Photos of a superlattice film peeled off from the substrate after assembly. The largest dimension of the assembled superlattice is 7 mm, which is a factor of about 100 000 larger than the nanocrystal edge length. Scale bar: 100 nm.

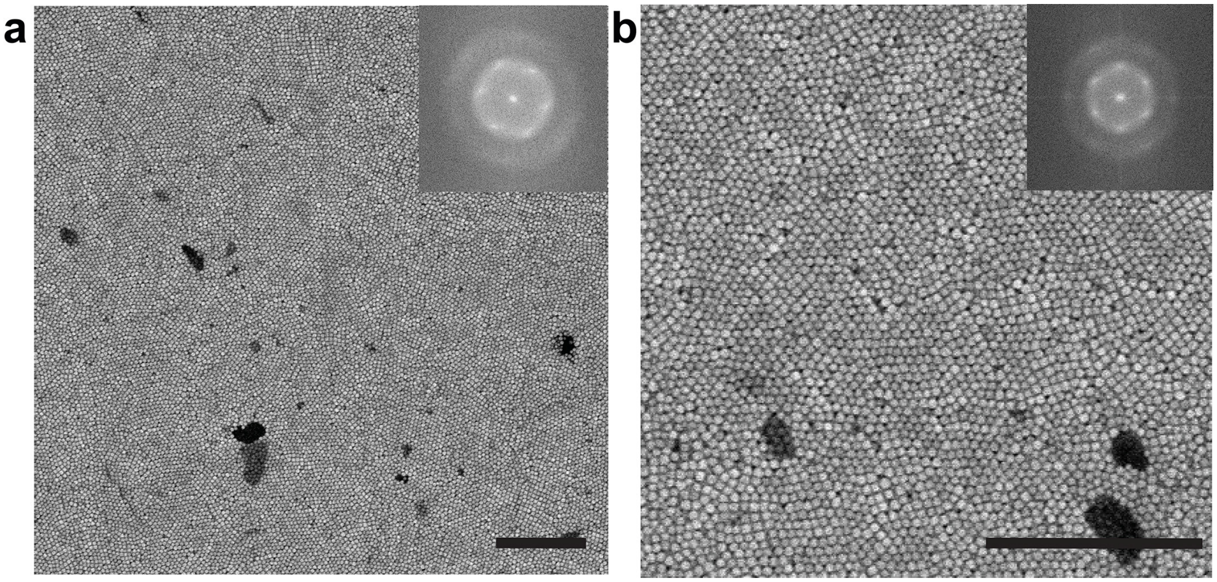

Supplementary Figure 2. SEM images of sphere superlattices at low (a) and high (b) magnifications. The micrographs are taken from the direction of the bottom superlattice surface, which had been in contact with the glass cuvette. The sphere superlattices consist of ordered domains with coherence over only a few microns. The insets show surface layer diffraction patterns obtained via a fast Fourier transform of the corresponding SEM images. Scale bar: 1 μm.

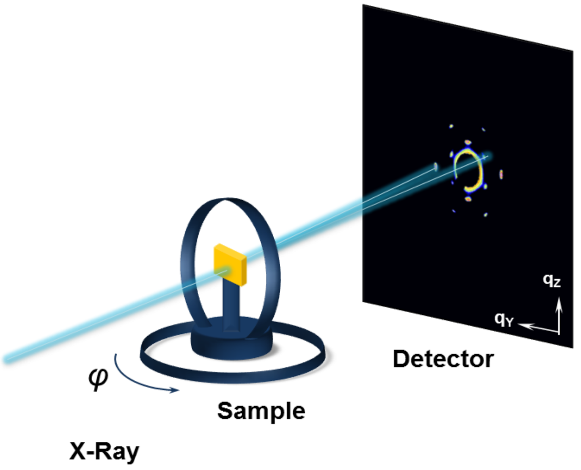

Supplementary Figure 3. Illustration of the geometric setup in SAXS experiments.

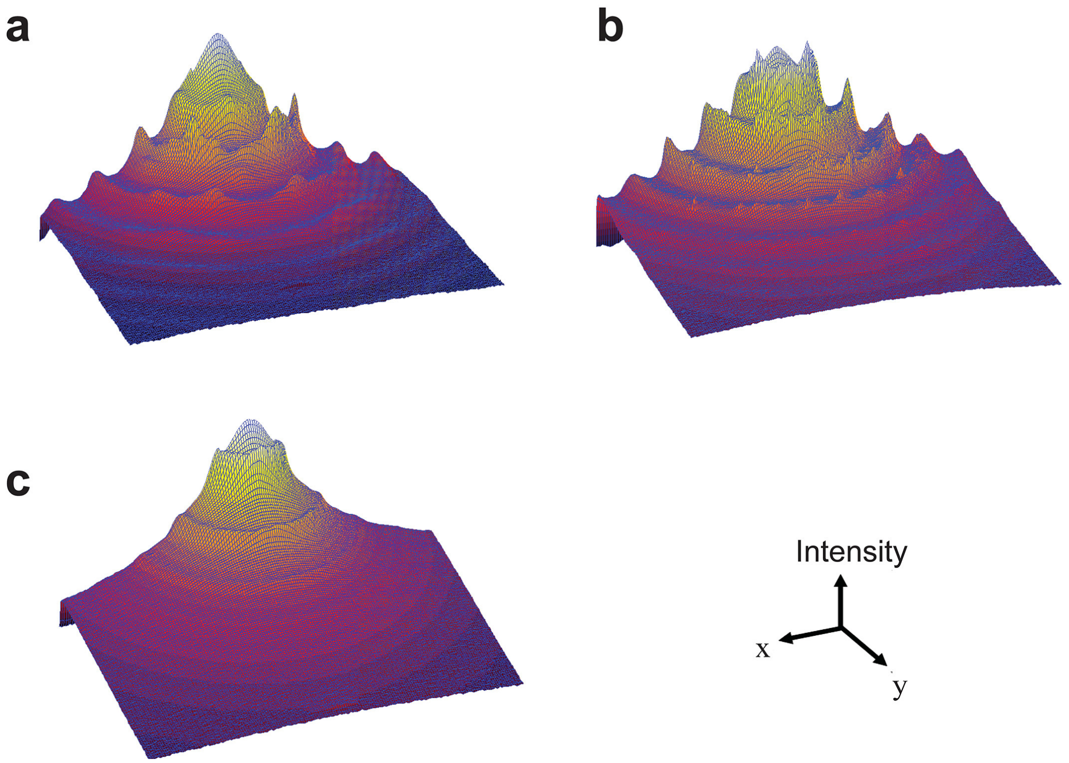

Supplementary Figure 4. Three-dimensional graphical representation of the SAXS intensity data for superlattices of (a) sRD, (b) octahedron, and (c) cube. Diffraction peaks are clearly visible for sRD and octahedron, but smeared out for cube.

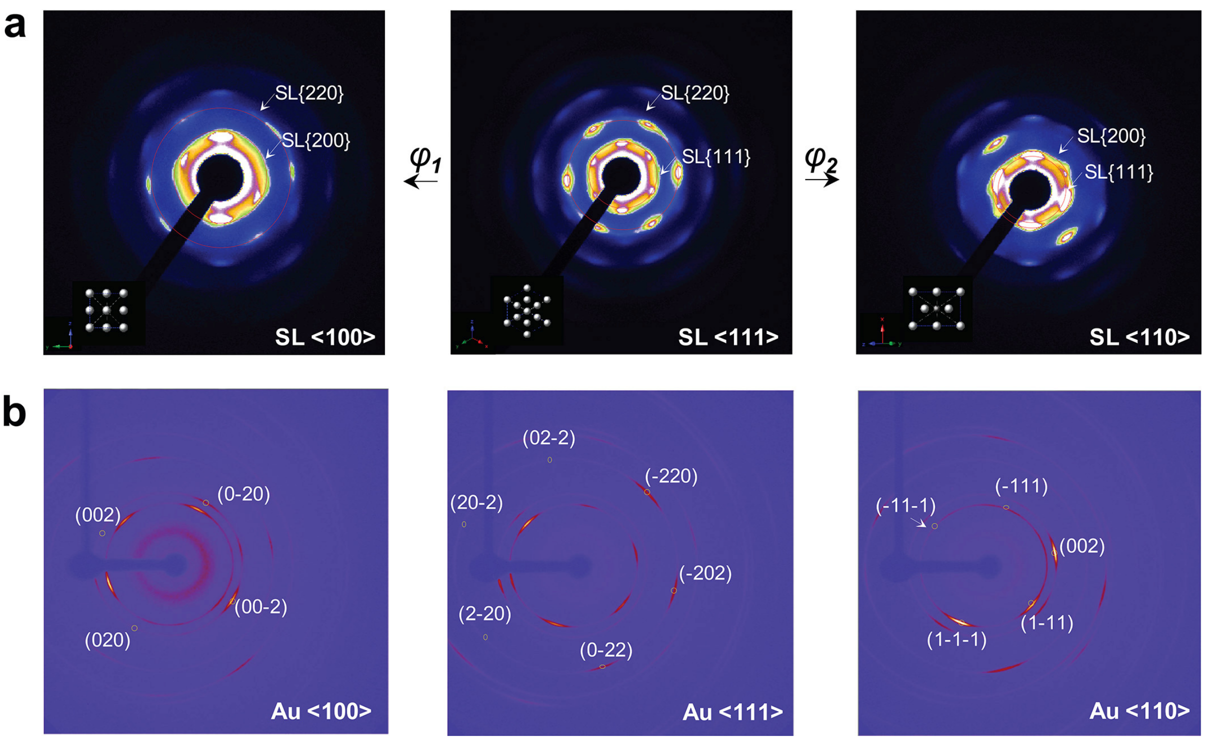

Supplementary Figure 5. SAXS (a) and single crystal X-ray diffraction (b) images of sRD superlattices obtained by rotating the sample. (a) In rotating SAXS, after finding the direction $<111>_{SL}$ of the superlattice (middle), the sample is rotated by $\varphi_1 = -35.26°$ into $<100>_{SL}$ (left) and $\varphi_2 = 54.73°$ into $<110>_{SL}$ (right). Clear diffraction spots corresponding to the face-centered cubic crystal indicate a single crystalline superlattice throughout the whole observed sample. (b) The sample is rotated and different orientations are shown: from left to right are <100>, <111> and <110> of Au, respectively.

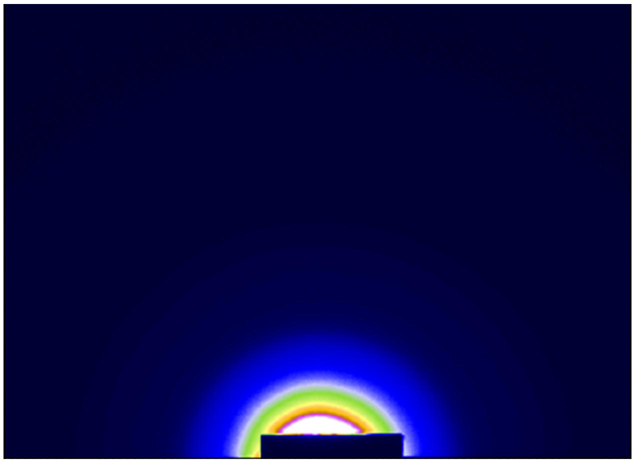

Supplementary Figure 6. SAXS data for assembled gold nanocrystal spheres, showing only isotropic scattering rings instead of diffraction spots.

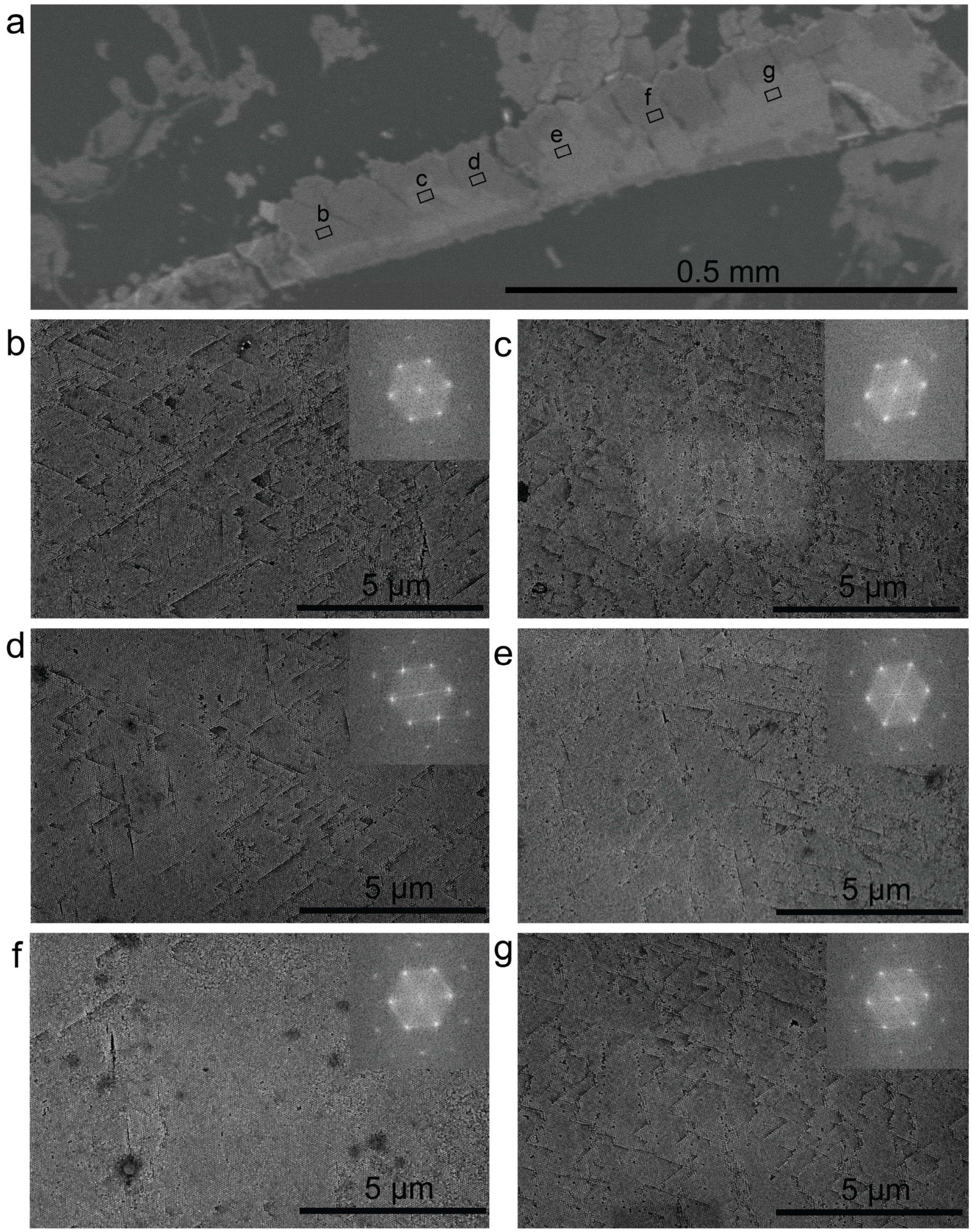

Supplementary Figure 7. (a) Low-magnification SEM image of a sRD sample. The largest dimension is longer than 0.5 millimeters. (b-g) Six higher-magnified images taken at various points along the sample. As visible in the fast Fourier transform patterns in regions marked in (a), the entire sample is single crystalline. Scale bar: (a) 0.5 mm; (b-g) 5 µm.

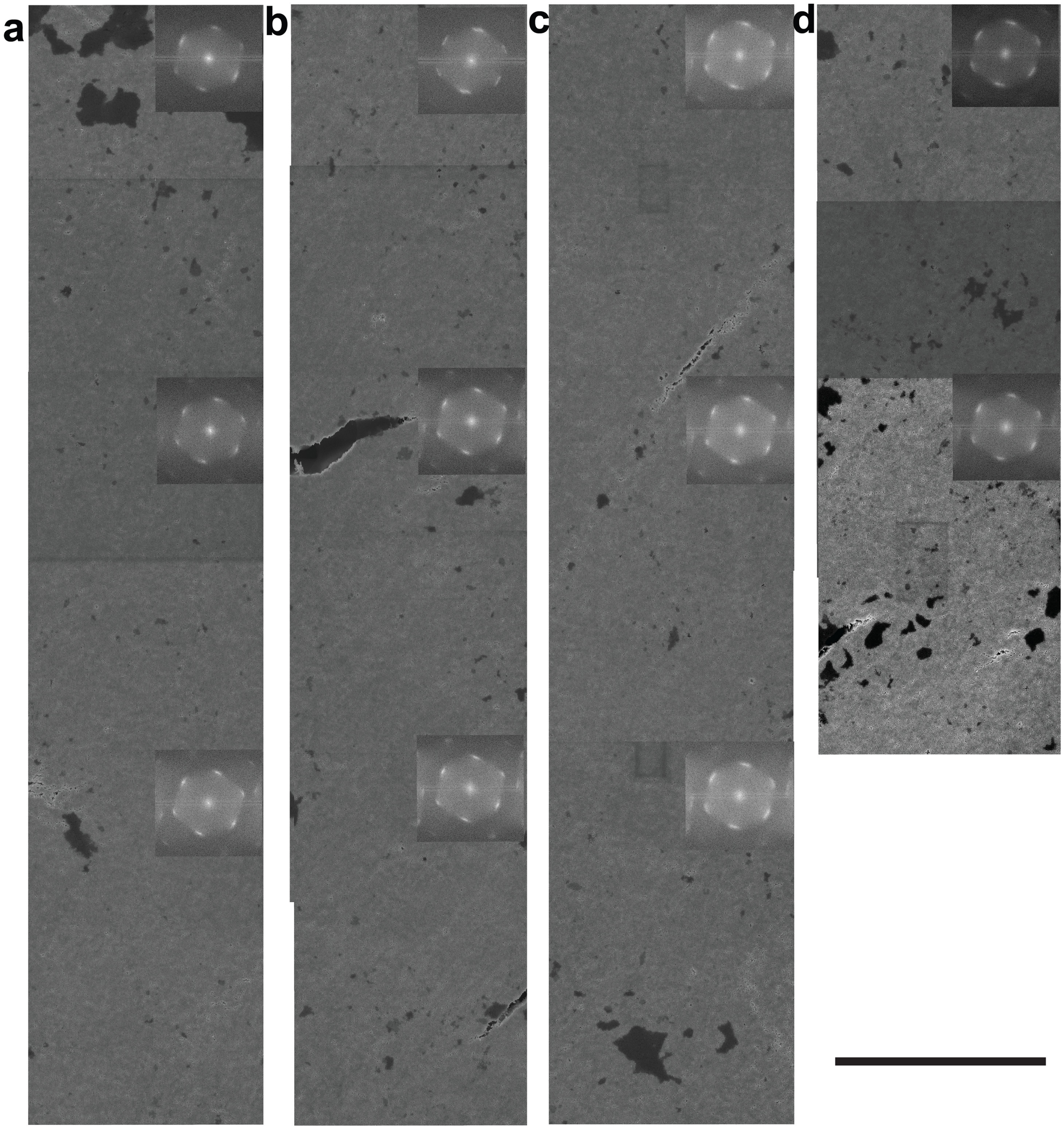

Supplementary Figure 8. Large-area panels of two continuous regions (a,b) and (c,d) of sRD superlattices stitched together from individual SEM images. Upper edges of (b) and (d) are the bottom edges of (a) and (c), individually. The consistent orientation of the fast Fourier transform pattern calculated locally for each snapshot demonstrates that both sample areas are single crystals. The domain size measured from top to bottom is at least 100 microns in (a, b) and at least 85 microns in (c, d). This distance comprises about 100 layers in both (a, b) and (c, d). Scale bar: 10 μm.

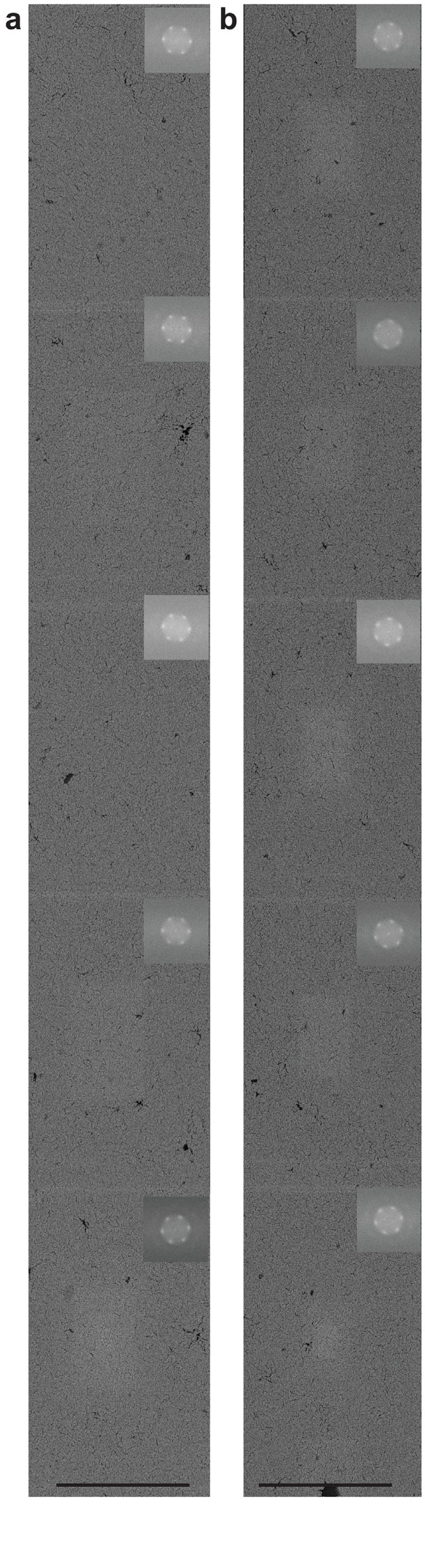

Supplementary Figure 9. Large-area panels of a mRD superlattice. (a) and (b) represent a continuous area stitched together from ten individual SEM images. The lower edge of (a) is connected with the upper edge of (b). In each snapshot the surface layer diffraction pattern (fast Fourier transform) is calculated locally. The orientation of the diffraction pattern remains constant except for a rapid change in the bottom of (a). This change is caused by the grain boundary. The larger single crystalline grain extends over at least 200 microns and comprises at least 100 layers. Scale bar: 10 μm.

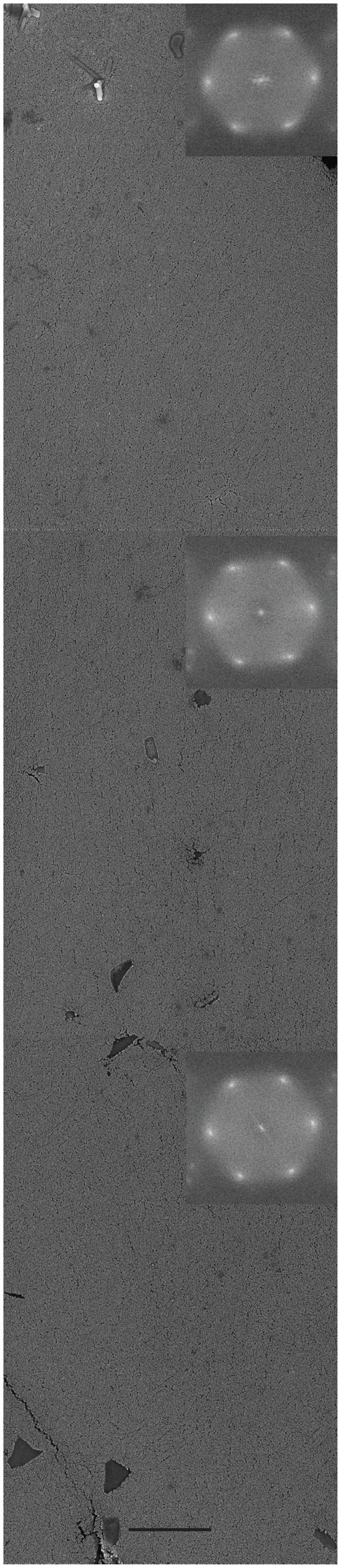

Supplementary Figure 10. Large-area panels of a lRD superlattice stitched together from three individual SEM images. The orientation of the surface layer diffraction pattern remains constant throughout the sample. The single crystalline grain extends over at least 190 microns and comprises at least 100 layers. Scale bar: 10 μm.

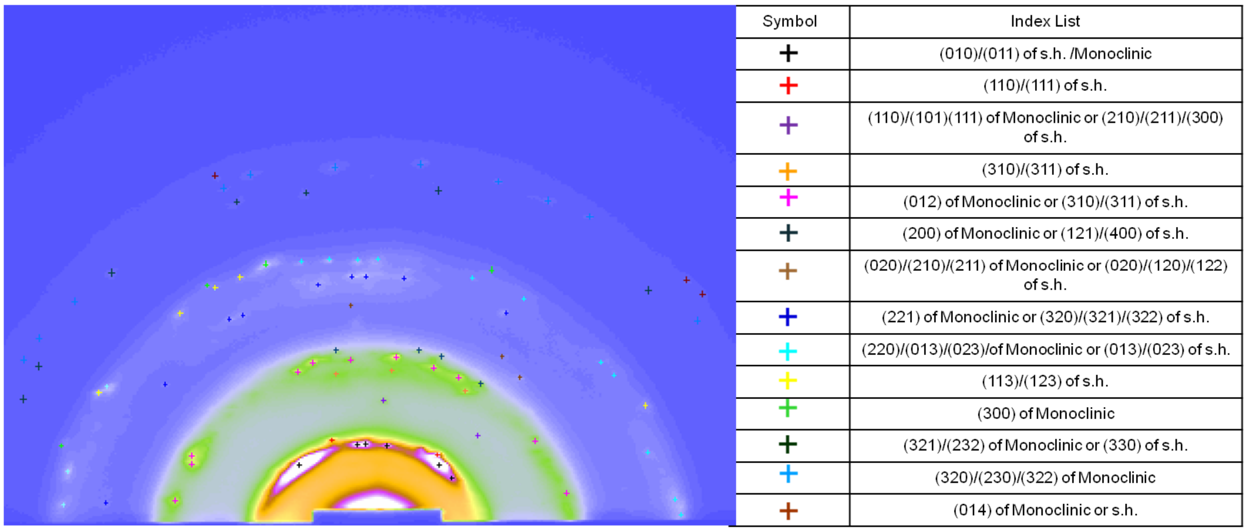

Symbol	Index List
+	(010)/(011) of s.h. /Monoclinic
+	(110)/(111) of s.h.
+	(110)/(101)(111) of Monoclinic or (210)/(211)/(300) of s.h.
+	(310)/(311) of s.h.
+	(012) of Monoclinic or (310)/(311) of s.h.
+	(200) of Monoclinic or (121)/(400) of s.h.
+	(020)/(210)/(211) of Monoclinic or (020)/(120)/(122) of s.h.
+	(221) of Monoclinic or (320)/(321)/(322) of s.h.
+	(220)/(013)/(023)/of Monoclinic or (013)/(023) of s.h.
+	(113)/(123) of s.h.
+	(300) of Monoclinic
+	(321)/(232) of Monoclinic or (330) of s.h.
+	(320)/(230)/(322) of Monoclinic
+	(014) of Monoclinic or s.h.

Supplementary Figure 11. Indexing of the peaks in the octahedron superlattice SAXS data. Peaks cannot be indexed using a single lattice type. Instead, we have to assign peaks to two lattices, a simple hexagonal lattice (s.h.) and a monoclinic lattice. The lattices are shown in Fig. 2e-h of the main text.

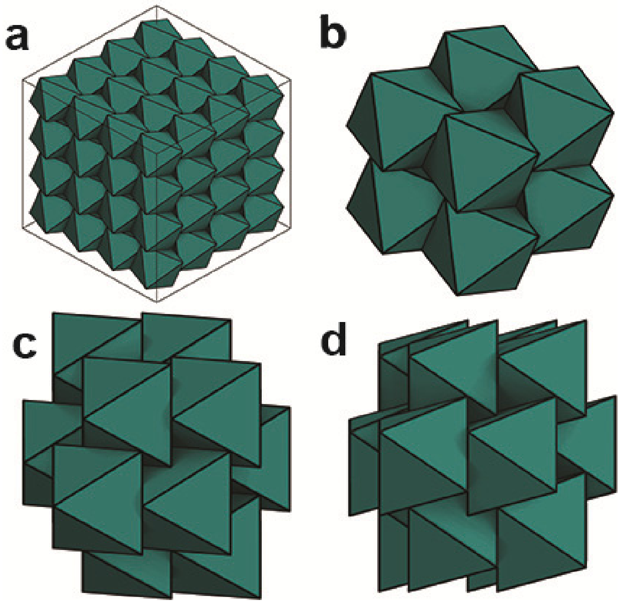

Supplementary Figure 12. Minkowski lattice, the densest packing of octahedra. (a) 4 × 4 × 4 array of the Minkowski lattice. (b-d) Single octahedron surrounded by its first shell of adjacent octahedra, observed under three different projections.

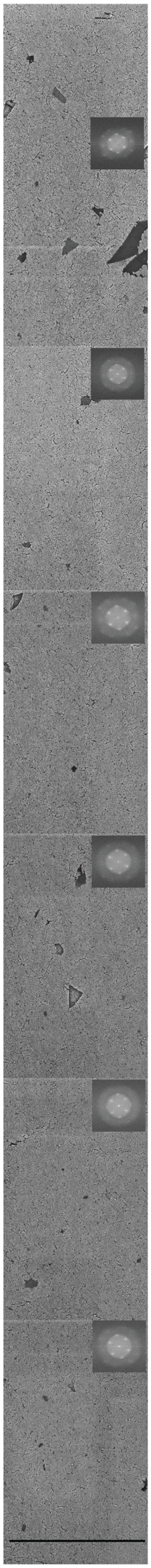

Supplementary Figure 13. Large-area panels of an octahedron superlattice stitched together from several individual SEM images. The orientation of the surface layer diffraction pattern remains constant throughout the sample. The single crystalline grain extends over at least 100 microns and comprises at least 50 layers. Scale bar: 10 μm.

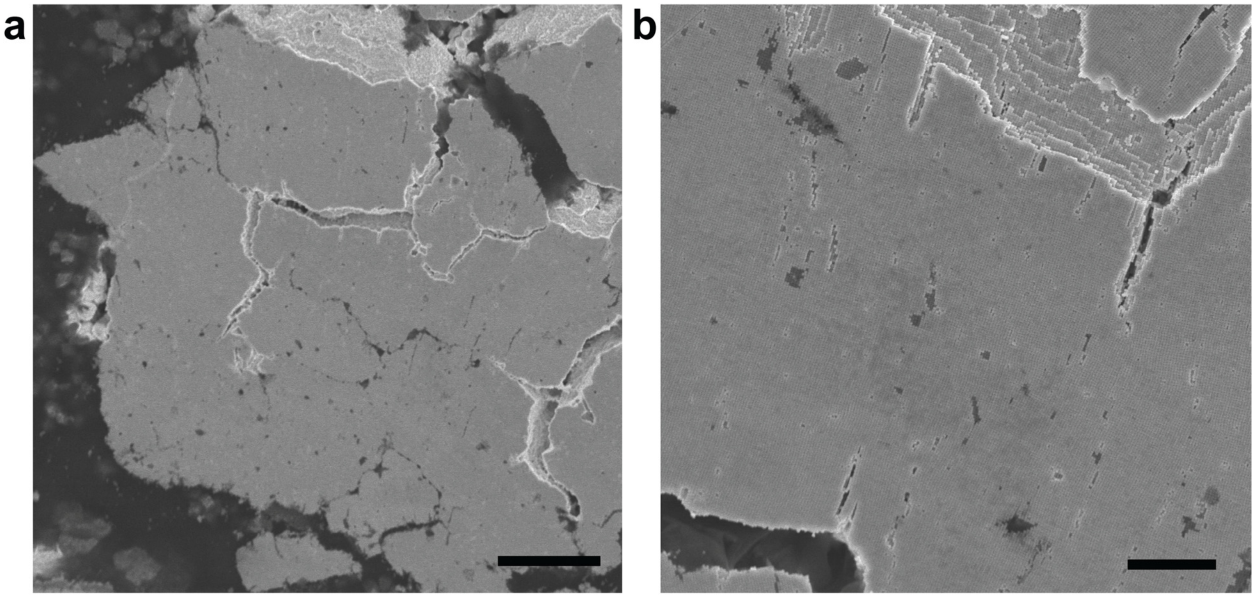

Supplementary Figure 14. Low-magnification SEM images of cube superlattices. The single crystalline domain size extends over at least 30 microns and comprises at least 50 layers. Scale bar: (a) 10 μm; (b) 2 μm.

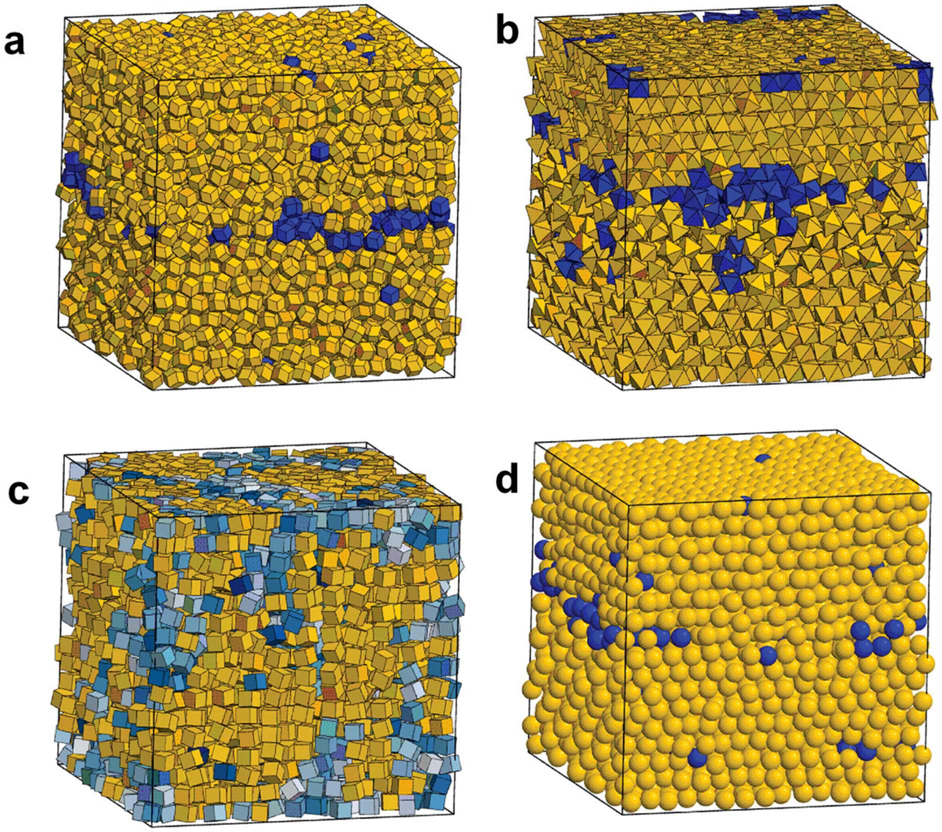

Supplementary Figure 15. Monte Carlo simulations with impenetrable hard boundaries at the top and bottom (in the image) of the simulation box to study heterogeneous nucleation. The sides are connected via periodic boundary conditions. Simulations are conducted for systems of (a) rhombic dodecahedra, (b) octahedra, (c) cubes, and (d) spheres. The crystal structure of the superlattices observed near the wall is identical to the crystal structure observed in nuclei forming via homogenous nucleation with the exception of octahedra, which prefer the monoclinic superlattice near the wall. The packing fractions used in the simulations are 0.56 for rhombic dodecahedra, 0.56 for octahedra, 0.55 for cubes, and 0.54 for spheres.

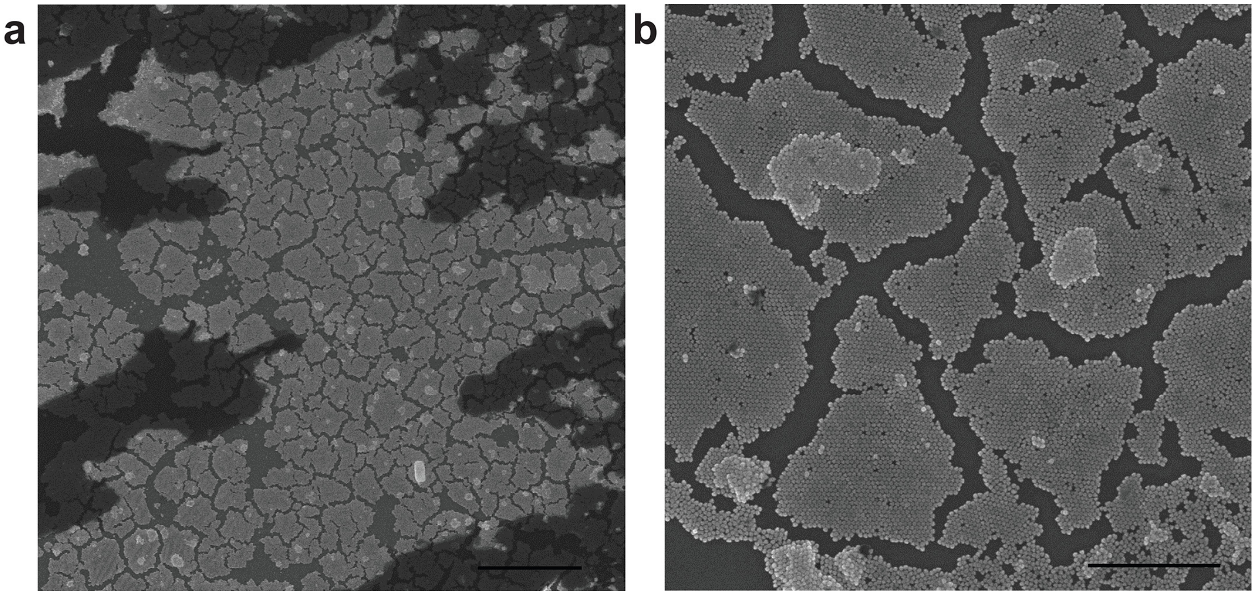

Supplementary Figure 16. Octahedron superlattices obtained at a significantly lower nanocrystal concentration than that of bulk superlattice formation. Octahedra still form dense layers of hexagonal packing. Single layers with a small portion of double layer are also observed. Scale bar: (a) 10 µm; (b) 2 µm.

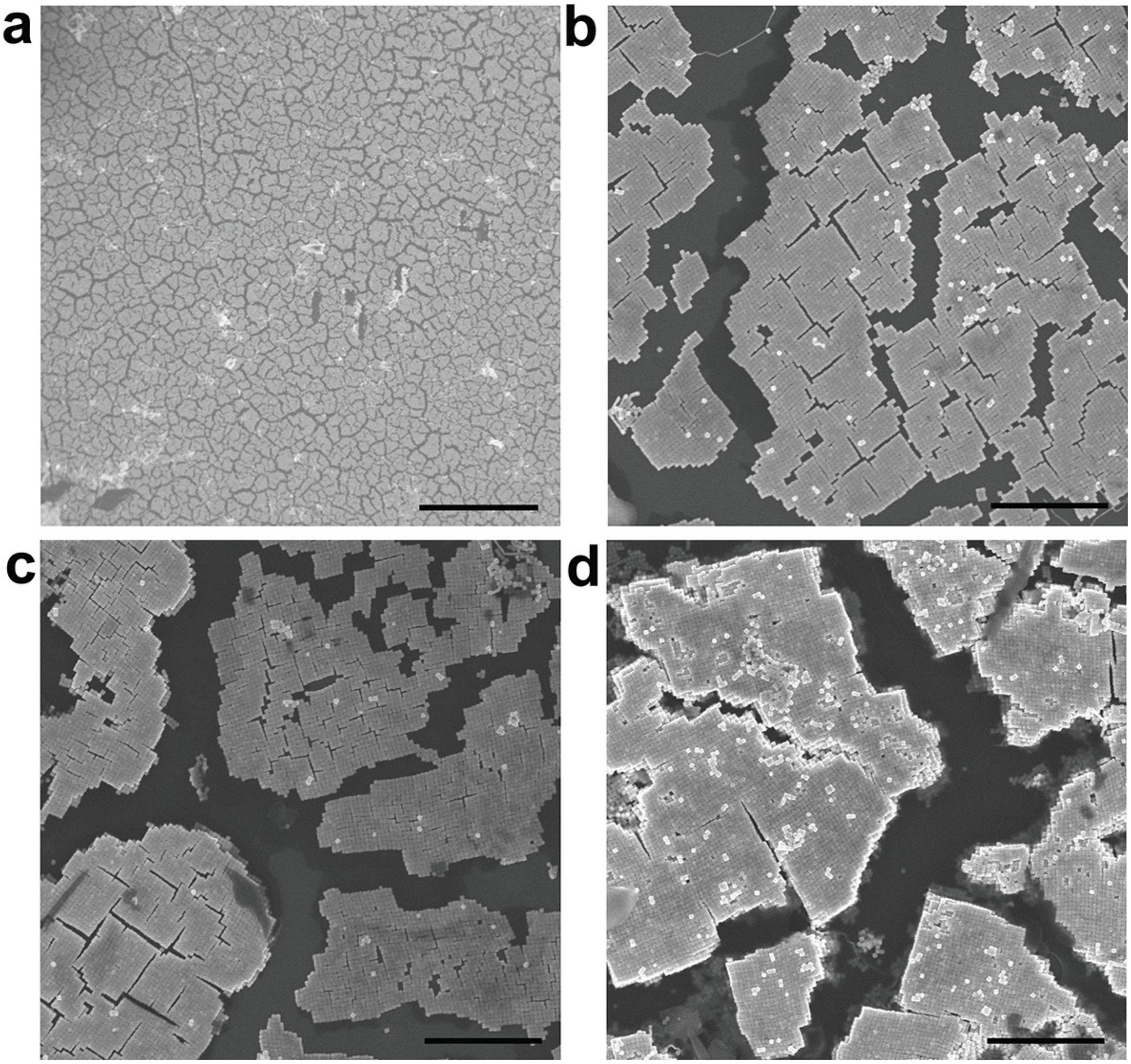

Supplementary Figure 17. Cube superlattices at a significantly lower nanocrystal concentration than that of bulk superlattice formation. (a) Large-scale view of the sample, (b) zoom-in on a single layer, (c) single layer (right part) and double layers (left part), (d) multi-layered superlattices. Scale bar: (a) 50 μm; (b-d) 2 μm.

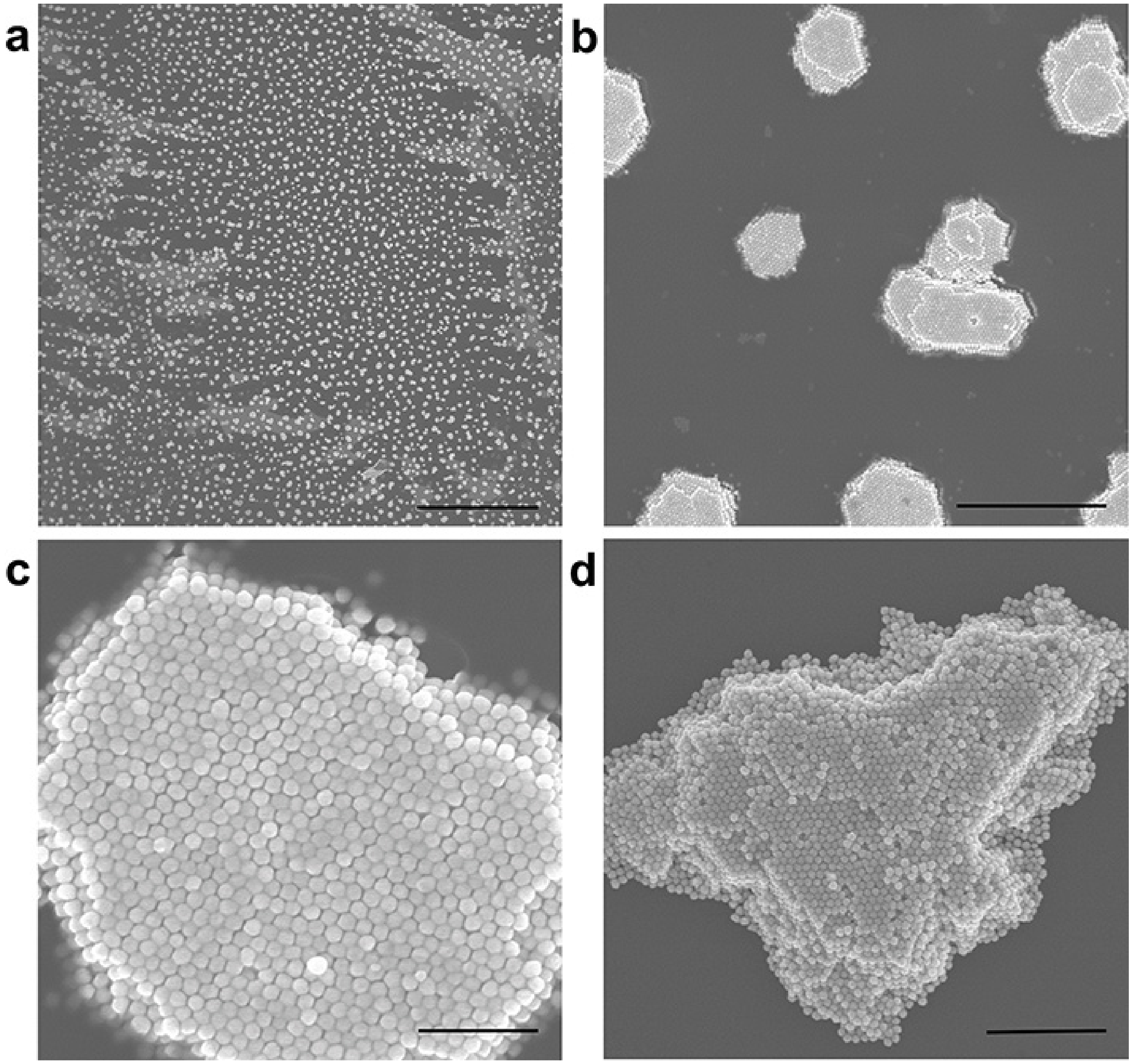

Supplementary Figure 18. Rhombic dodecahedron superlattices obtained at a significantly lower nanocrystal concentration than that of bulk superlattice formation. Nanocrystals assemble into small clusters spanning a few tens of particle diameters. Scale bar: (a) 50 µm; (b) 5 µm; (c) 500 nm; (d) 100 nm.

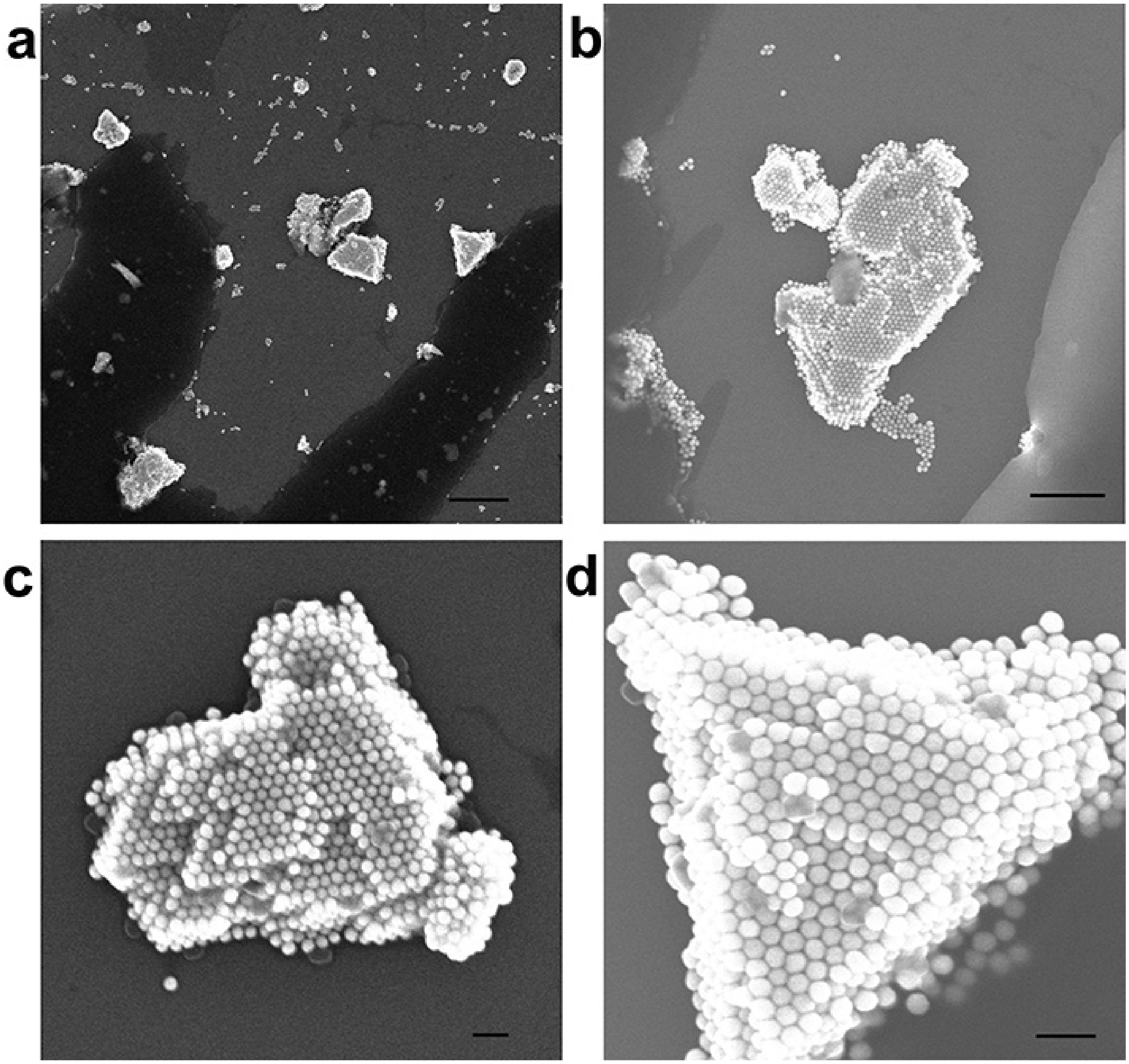

Supplementary Figure 19. Sphere superlattices obtained at a significantly lower nanocrystal concentration than that of bulk superlattice formation. Nanocrystals assemble together into small clusters spanning a few tens of particle diameters. Scale bar: (a) 2 µm; (b) 1 µm; (c) 200 nm; (d) 200 nm.

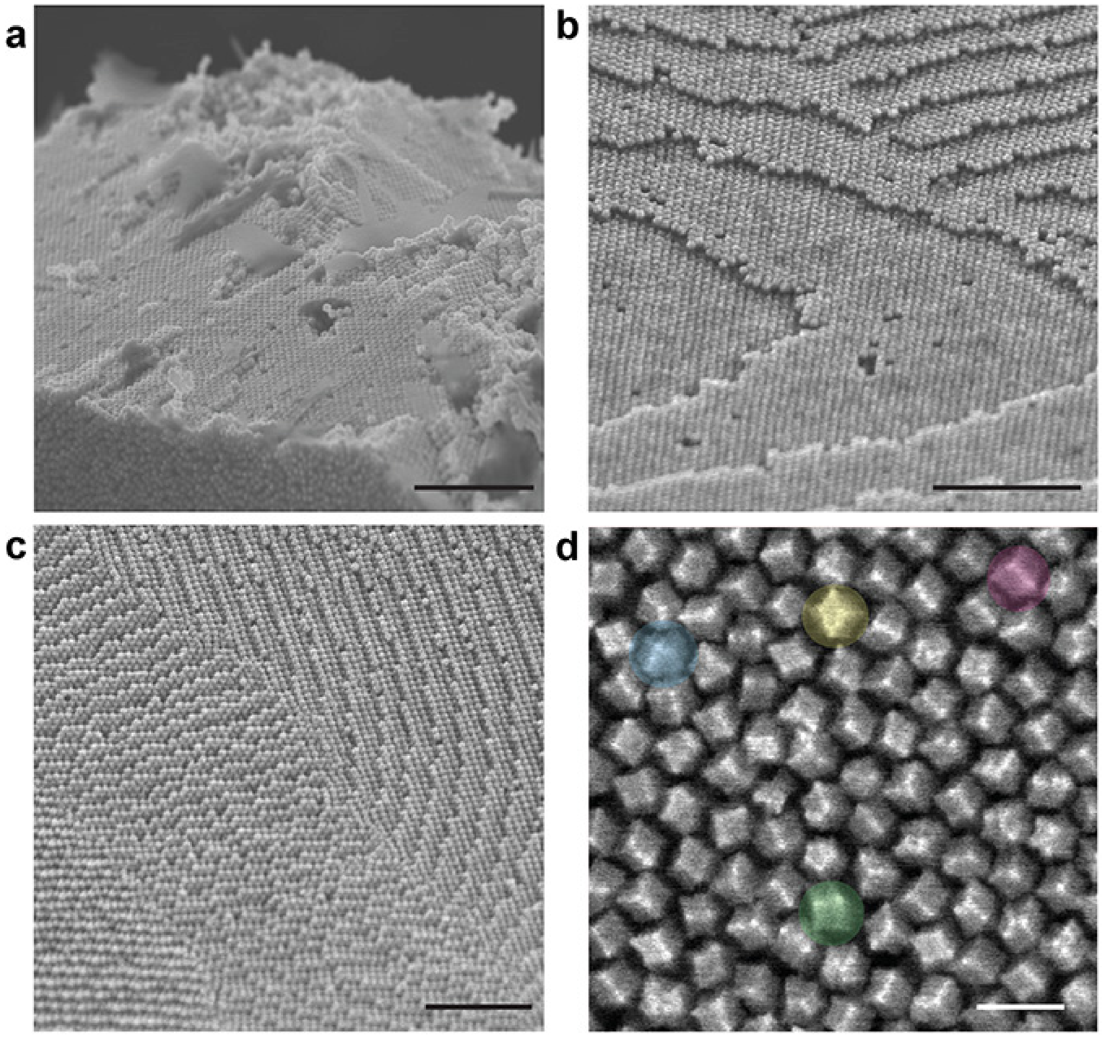

Supplementary Figure 20. Additional SEM images of mRD superlattices from other directions rather than the bottom of glass cuvette, in which they were grown. (a) and (b) lateral views, (c) surface exposed by breaking the superlattice, (d) evidence for a frozen in rotator phase in mRD superlattices. Typical example defects are highlighted in colors: 4-fold vertex (blue), face (yellow), edge up (purple), and improper $\pi/6$ rotation of a 3-fold vertex (green). Scale bar: (a, b) 500 nm; (c) 1 μm; (d) 200 nm.

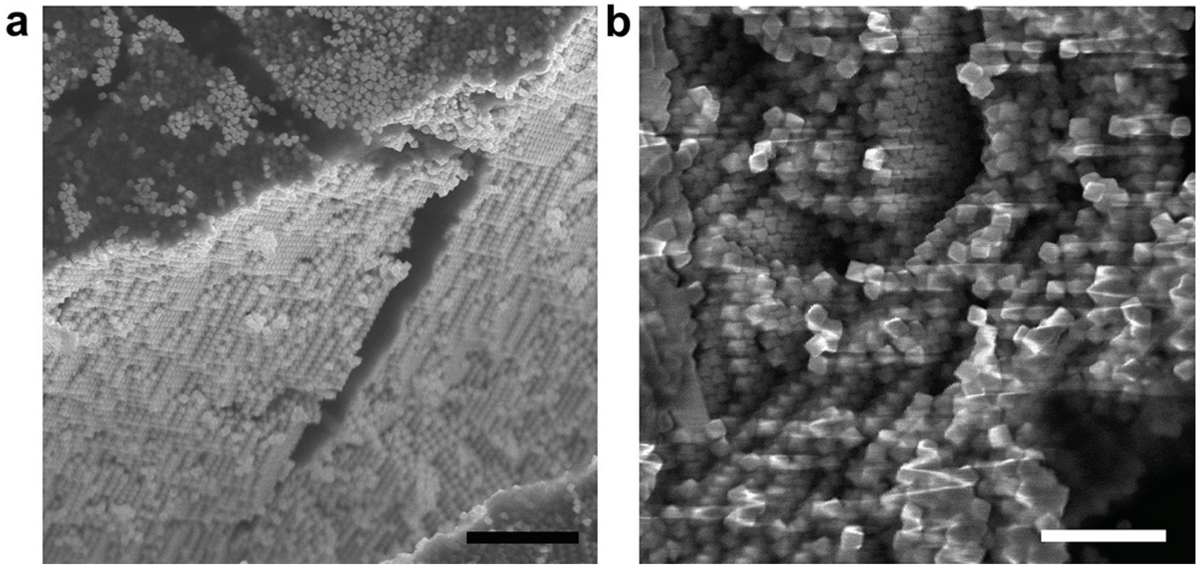

Supplementary Figure 21. SEM images of octahedron superlattices from other directions rather than the bottom of glass cuvette, in which they were grown. (a) Cross section and (b) lateral view. Splitting hexagonal layers are exposed by fracturing, which occurred during drying of the sample (b). Scale bar: (a) 1 μm; (b) 500 nm.

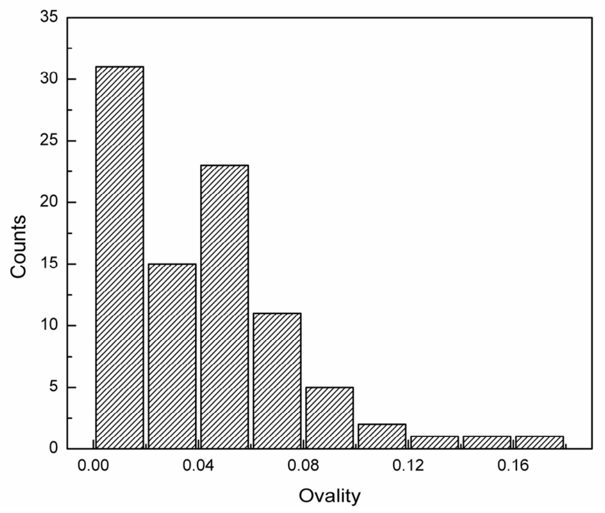

Supplementary Figure 22. Histogram of the ovalities of 100 randomly selected as-synthesized gold nanospheres. Ovality is defined as $O = 2(a - b)/(a + b)$, where a and b are the major and minor axis of the nanosphere, respectively. The ovality of 80% of the nanospheres is below 0.06 and the average ovality is $\overline{O} = 0.038$.
